# Interventional radiology knowledge among family medicine residents in Türkiye: a national cross-sectional survey

**DOI:** 10.1186/s12909-026-09916-y

**Published:** 2026-07-11

**Authors:** Süveybe PEKER, Mustafa GÖK, Serpil DEMİRAĞ

**Affiliations:** 1 Department of Family Medicine, Aydin Adnan Menderes University, Faculty of Medicine, Zafer Mah., 09100 Aydin, Türkiye; 2 Department of Radiology, Section of Interventional Radiology, Aydin Adnan Menderes University, Faculty of Medicine, Zafer Mah., 09100 Aydin, Türkiye

**Keywords:** Interventional radiology, Family medicine residents, Knowledge, Medical education, Referral patterns, Cross-sectional study

## Abstract

**Background:**

Interventional radiology (IR) provides minimally invasive diagnostic and therapeutic procedures and is increasingly integrated into routine clinical care. Family physicians play a key role in recognizing indications for IR and referring patients appropriately; however, their level of knowledge regarding IR remains uncertain.

**Aims:**

To assess the knowledge, awareness, and perceptions of interventional radiology among family medicine residents in Türkiye and to evaluate factors associated with knowledge levels.

**Methods:**

This cross-sectional analytical study was conducted among family medicine residents between August and October 2023 using a structured 21-item online questionnaire. The survey assessed sociodemographic characteristics, exposure to IR training, and knowledge of IR procedures. Knowledge scores were calculated using ten items. Statistical analyses were performed using descriptive statistics, t-tests, ANOVA, and chi-square tests, with significance set at *p* < 0.05.

**Results:**

A total of 413 residents participated in the study. Overall, 60.6% of participants reported having an adequate level of knowledge regarding interventional radiology (IR), whereas only 25.5% stated that they directly referred patients to IR units. Commonly performed procedures, such as image-guided biopsy and drainage, were correctly identified by most participants; however, misconceptions were noted regarding several other interventional procedures. Residents who had completed clinical rotations associated with IR, particularly in cardiology and obstetrics and gynecology, demonstrated higher knowledge scores compared with those without such experience.

**Conclusions:**

Family medicine residents demonstrated moderate awareness of interventional radiology, with knowledge gaps remaining in specific procedural areas. Clinical exposure through rotations may improve understanding. Incorporating structured IR education into residency training may enhance appropriate referral practices.

**Supplementary Information:**

The online version contains supplementary material available at 10.1186/s12909-026-09916-y.

## Introduction

Interventional radiology (IR) is a specialized subspecialty of radiology that performs percutaneous or intravascular procedures using various imaging modalities and provides minimally invasive alternatives to conventional surgical interventions. These procedures serve both diagnostic and therapeutic purposes and are generally associated with lower complication rates and shorter recovery times. Interventional radiology provides care for a broad spectrum of patients, ranging from pediatric to geriatric populations, from outpatients to critically ill intensive care patients, and from individuals with infections to those undergoing chemotherapy [1–7]. Since its emergence in the 1960s, the scope and diversity of IR procedures have expanded considerably [7, 8]. Today, a wide variety of procedures, including vascular interventions, oncologic treatments, drainage procedures, biopsies, and embolization therapies, are performed by interventional radiologists. However, with the increasing importance of endovascular and minimally invasive procedures, image-guided interventions are now performed not only by radiologists but also by specialists from various disciplines, including cardiology, cardiovascular surgery, gastroenterology, neurosurgery, and anesthesiology and reanimation.

Family physicians (FPs) play a pivotal role in providing comprehensive and continuous care and serve as the first point of contact within the healthcare system, particularly in Türkiye [9]. In primary care, FPs are expected to develop continuous partnerships with their patients, address individual health needs, consider the family and community context, and remain easily accessible to patients [10]. Within this framework, FPs are often the first physicians consulted by patients and play a critical role in referring patients to hospitals and specialized departments, as well as presenting potential treatment options. Therefore, it is crucial for FPs to have sufficient knowledge of IR and the various alternative treatment modalities available for different disease processes to ensure optimal patient management [10].

A study conducted in Canada in 2010 involving 213 family physicians found that 53% of participants considered their knowledge adequate, 98% were aware that interventional radiologists perform image-guided biopsies, and approximately 71% referred patients directly to interventional radiologists [11]. In a more recent study conducted in Saudi Arabia in 2023 with 197 participants, only 28.4% were found to have a generally good level of knowledge regarding IR [12]. Although limited, existing literature suggests that family physicians may have insufficient awareness of IR. Given the rapid advancements and increasing utilization of IR, FPs are integral in educating patients and directing them to appropriate specialists [13].

Despite the growing significance of IR in modern medicine, the awareness and understanding of this specialty among family physicians remain limited. This gap in knowledge may hinder timely referrals and optimal patient care. To date, no studies have evaluated the level of IR knowledge among family medicine residents in Türkiye, a frontline group responsible for patient guidance and coordination of care. The present study therefore aims to assess the knowledge and awareness of IR among family medicine residents in Türkiye and to identify potential educational gaps that may inform future training strategies.

## Materials and methods

The study was designed as a cross-sectional analytical study, aimed at assessing the opinions, experiences, and knowledge levels of family medicine residents regarding IR. Our research was approved by the local institutional ethics committee (Protocol No: 2023/149; Approval No: E-53043469-050.04.04-390281).

The questionnaire was developed following a comprehensive review of the existing literature and was inspired by validated questionnaires used in previous studies addressing similar topics. The final version of the questionnaire was refined after pilot testing in a small group of 25 participants, incorporating feedback from the pilot process as well as revisions and expert opinions provided by SD, a family medicine specialist with 30 years of clinical experience, and MG, an interventional radiologist with 15 years of clinical experience. The questionnaire used in this study was originally designed by the researchers based on previous literature and expert opinions (see Supplementary File 1).

The questionnaire consisted of 21 structured items evaluating participants’ sociodemographic characteristics as well as various dimensions of their knowledge and perceptions regarding interventional radiology (IR). Among these 21 items, 10 were specifically designed to assess knowledge level, with each correct response assigned 1 point. Knowledge level was evaluated on a 10-point scale, and participants scoring 6 points or higher were considered to have an adequate level of knowledge. The questions were designed to assess not only awareness but also knowledge regarding specific IR procedures and their applications in clinical practice.

The target population included all family medicine residents actively practicing in Türkiye during the data collection period, from August 1 to October 31, 2023. Sample size determination was performed using Open-Epi version 2013, an open-source epidemiological statistics tool developed by Dr. Will (Bill) J. Farland at Emory University. Based on the total number of residents, a minimum sample size of 346 was calculated to achieve adequate statistical power. To account for possible non-response and exclusion criteria, the target was increased by 10%, resulting in a final desired sample size of at least 381 participants.

The questionnaire was administered exclusively online through Google Forms, a secure platform governed by the laws of the State of Delaware, USA, and compliant with applicable U.S. regulations. The survey link was disseminated via electronic mailing lists, individual emails, and social media channels targeted specifically at family medicine residents. To minimize response bias and duplicate entries, the questionnaire was shared only once with each participant. Participation was voluntary, and informed consent was obtained electronically by requiring respondents to check an approval box before proceeding. Personal identifiers were not collected, ensuring anonymity and confidentiality throughout the study.

A total of 413 family medicine residents completed the survey in full and were included in the final analysis. Data were analysed using IBM SPSS Statistics version 25.0 (IBM Corp., Armonk, NY, USA). Descriptive statistics were calculated for all variables. The Independent Samples t-test was used to compare means between two groups, while One-Way Analysis of Variance (ANOVA) was applied for comparisons involving three or more groups, with Bonferroni post-hoc corrections to identify specific group differences when necessary. Categorical variables were analysed using the Chi-square test. Statistical significance was set at *p* < 0.05.

## Results

The sociodemographic and professional characteristics of the resident physicians are detailed in Table 1.

**Table 1:** Frequency distribution of demographic and various characteristics

Variables		*n*	%
Gender			
Female	302		73.1
Male	111		26.9
Years in Profession Mean ± SD (median, min-max)	7.59 ± 7.49 (5.1–48)		
≤5 years	224		56.3
6–10 years	87		21.9
>10 years	87		21.9
Year of Residency			
1st year	156		38.0
2nd year	126		30.7
3rd year	76		18.5
4th year and beyond (CFMS)	52		12.7
Institution of Residency			
University hospital	346		84.2
Training and research hospital	65		15.8
Type of Residency Program			
Family Medicine Specialty (FMS)	260		63.4
Contracted Family Medicine Specialty (CFMS)	150		36.6

*FMS:* Family Medicine Specialty,  *CFMS:* Contracted Family Medicine Specialty

When evaluating whether participants had completed their mandatory and elective rotations, it was found that 81.0% (*n* = 333) had not completed the rotation process.

A detailed evaluation of the participants’ rotation status and their level of knowledge regarding interventional radiology is presented in Table 2.

**Table 2:** Level of knowledge of interventional radiology according to clinical rotations

Rotations		Knowledge level regarding interventional radiology			
		Good (*n* = 57)	Moderate (*n* = 194)	Poor (*n* = 162)	*p*
Completion of Rotations	Yes	14 (24.6)	34 (17.5)	31 (19.1)	0.494
	No	43 (75.4)	160 (82.5)	131 (80.9)	
Completion status of clinical rotations					
Internal Medicine	No	21 (36.8)	77 (39.7)	69 (42.6)	0.718
	Yes	36 (63.2)	117 (60.3)	93 (57.4)	
Paediatrics	No	24 (42.1)	78 (40.2)	89 (54.9)	0.017
	Yes	33 (57.9)	116 (59.8)	73 (45.1)	
Obstetrics Gynaecology	No	24 (42.1)	81 (41.8)	75 (46.3)	0.670
	Yes	33 (57.9)	113 (58.2)	87 (53.7)	
Elective Rotations	No	26 (45.6)	98 (50.5)	92 (56.8)	0.276
	Yes	31 (54.4)	96 (49.5)	70 (43.2)	
Psychiatry	No	30 (52.6)	117 (60.3)	92 (56.8)	0.551
	Yes	27 (47.4)	77 (39.7)	70 (43.2)	
Dermatology	No	26 (45.6)	108 (55.7)	95 (58.6)	0.234
	Yes	31 (54.4)	86 (44.3)	67 (41.4)	

When participants were asked about their level of knowledge regarding interventional radiology (IR), 60.6% (*n* = 249) reported that they had an adequate level of knowledge.

When asked about sources of knowledge regarding interventional radiology (IR), 81.3% of participants (*n* = 334) reported that they had acquired their knowledge about IR during their medical school education.

Regarding participants’ perceptions of interventional radiologists’ training background, 93.4% (*n* = 383) believed that interventional radiologists complete diagnostic radiology residency training, and 96.6% (*n* = 396) thought that they receive formal training in image-guided interventional procedures.

In terms of referral practices, 25.5% of participants (*n* = 107) reported that they directly referred patients to IR units following elective procedures.

The responses provided by participants regarding procedures performed by Interventional Radiology are presented in Table 3.

**Table 3:** Frequency distribution of responses regarding procedures performed by interventional radiology

Procedures Performed by Interventional Radiology	Correct, *n* (%)	Incorrect/Don’t Know, *n* (%)
1. Biopsy or drainage using imaging techniques	393 (95.2)	20 (4.8)
2. Cardiac angioplasty or stenting	131 (31.7)	282 (68.3)
3. Vascular angioplasty procedures	299 (72.4)	114 (27.6)
4. Lower extremity vascular bypass procedures	138 (33.4)	275 (66.6)
5. Epistaxis (nose-bleeding) management	158 (38.3)	255 (61.7)
6. Uterine artery embolization for symptomatic uterine fibroids	278 (67.3)	135 (32.7)
7. Vacuum-assisted excision of breast fibroadenoma	222(53.8)	191(46.2)
8. Pelvic congestion syndrome	259(62.7)	154(37.3)
9. Placement of percutaneous nephrostomy catheter in renal collecting systems	320(77.5)	93(22.5)
10. Clipping of cerebral vascular aneurysm	291(70.5)	122(29.5)

The Cronbach’s alpha value for the ten items aimed at measuring knowledge level was determined to be 0.51 [14]. The average total score for the IR knowledge questions was calculated as 6.34.

The relationship between knowledge level regarding Interventional Radiology and completed clinical rotations is detailed in Table 4.

**Table 4:** Total interventional radiology knowledge scores according to completed rotations

Variables		*n*	Total Interventional Radiology Knowledge Score Mean ± SD	t	*p*
Completion Status of Rotations	**Yes**	78	6.33 ± 1.96	-0.010	0.992
	**No**	333	6.34 ± 1.96		
Details of Rotations					
Internal Medicine	**No**	166	6.17 ± 1.87	-1.457	0.146
	**Yes**	246	6.46 ± 2.01		
Paediatrics	**No**	190	6.04 ± 1.94	-2.934	**0.004**
	**Yes**	222	6.60 ± 1.94		
Obstetrics Gynaecology	**No**	179	6.09 ± 1.96	-2.287	**0.023**
	**Yes**	233	6.54 ± 1.94		
Elective Rotations	**No**	215	6.18 ± 1.92	-1.779	0.076
	**Yes**	197	6.52 ± 1.99		
Psychiatry	**No**	238	6.22 ± 1.98	-1.487	0.138
	**Yes**	174	6.51 ± 1.93		
Dermatology	**No**	228	6.14 ± 1.95	-2.428	**0.016**
	**Yes**	184	6.60 ± 1.94		
Emergency Medicine	**No**	224	6.15 ± 2.01	-2.199	**0.028**
	**Yes**	188	6.57 ± 1.87		
Respiratory Medicine	**No**	232	6.15 ± 1.92	-2.247	**0.025**
	**Yes**	180	6.59 ± 1.99		
Cardiology	**No**	218	6.11 ± 1.98	-2.550	**0.011**
	**Yes**	194	6.60 ± 1.91		

Values in bold indicate statistical significance *p*<0.05

The effect of sociodemographic characteristics of the participants on total IR knowledge scores are detailed in Table 5.

**Table 5:** Effect of sociodemographic characteristics on total interventional radiologknowledge scores

Variables		(*n* = 413)	Total IR Knowledge Score (Mean ± SD)	t	F	*p*	Post-Hoc
*Gander*	Female	302	6,33 ± 1,99	-0,217		0,828	
	Male	111	6,38 ± 1,88				
Years in practice	≤ 5 years	224	6,41 ± 1,92		0,384	0,681	-
	6–10 years	87	6,21 ± 1,96				
	> 10 years	87	6,30 ± 2,07				
Year of residency training	1st year	156	6,34 ± 1,95		0,129	0,943	-
	2nd year	126	6,36 ± 2,09				
	3rd year	76	6,43 ± 1,92				
	≥ 4th year	52	6,21 ± 1,76				
Type of institution	University hospital	346	6,34 ± 1,98	-0,183		0,855	-
	Training and research hospital	65	6,38 ± 1,83				
Residency program type	Family medicine specialty(FMS)	260	6,44 ± 1,97	1,354		0,176	-
	Contracted Family Medicine Specialty(CFMS)	150	6,17 ± 1,93				

## Discussion

Analysis of the participants’ sociodemographic characteristics revealed that approximately three-quarters were female physicians, over half had been practicing medicine for five years or less, about one-third were in their first year of residency training, and the majority (around 80%) were working at university hospitals. These demographic features align closely with findings reported in other studies. For example, Algharras et al. (2024) reported that 41.6% of family physicians in Saudi Arabia had between two and five years of professional experience [12].

In the knowledge assessment items, 60.6% of participants (*n* = 249) in our study reported having an adequate level of knowledge regarding interventional radiology (IR) concepts and procedures. This finding is consistent with the overall score obtained from our specifically designed questionnaire (6.34/10). These results are also in line with the study by Mok et al., which reported that 53% of family physicians had an adequate level of knowledge [11]. In contrast, Algharras et al. reported an adequate knowledge level of 39.2% among medical students, suggesting that knowledge of IR increases with clinical experience [12]. Overall, these findings indicate that awareness of IR among family physicians is at a moderate level; however, it still requires further improvement.

Most of the participants reported acquiring their IR knowledge during medical school. This is echoed by Xu et al., whose study in the UK found that many residents and medical students were first exposed to IR concepts during clinical training [15]. This highlights the potential value of integrating IR topics into undergraduate curricula to build foundational knowledge early.

In our cohort, the referral rate to Interventional Radiology units—either directly or indirectly—was 25.5%. This rate is somewhat lower than that reported by Clements et al. (29.4%) but markedly lower than the 71.9% reported in a Canadian study by Mok et al. [11, 16]. Such variations may reflect differences in healthcare systems, awareness levels, and accessibility to IR services.

When assessing familiarity with specific IR procedures, nearly all participants correctly identified imaging-guided biopsies and drainage procedures. More than three-quarters also correctly recognized procedures such as percutaneous nephrostomy catheter placement and vascular angioplasty. However, approximately 75% incorrectly identified cardiac angioplasty and stenting as IR procedures. Comparable trends were observed in Mok et al.’s study, where 98% correctly identified image-guided biopsy, but recognition of other procedures such as uterine artery embolization (71%), radiofrequency ablation (47%), and vascular angioplasty (38%) was lower [11]. Algharras et al. reported similar findings, and Shaikh et al. demonstrated that educational interventions significantly improved knowledge of IR procedures [12, 17]. This difference may be due to recent advances in IR education or specific characteristics of our cohort.

One of the notable findings of our study was that family medicine residents who had completed rotations in pediatrics (*p* = 0.004), obstetrics and gynecology (*p* = 0.023), dermatology (*p* = 0.016), emergency medicine (*p* = 0.028), pulmonology (*p* = 0.025), and cardiology (*p* = 0.011) achieved significantly higher scores in knowledge assessments. This may be associated with the additional clinical exposure provided by these rotations. Residents without such rotation experience more frequently rated their IR knowledge level as “insufficient,” suggesting the potential benefit of practical clinical exposure. However, further studies are needed to confirm this association.

It should be noted that the scope of interventional radiology (IR) procedures may vary considerably between institutions and countries. This variability arises from the fact that many interventional procedures are performed not only by interventional radiologists but also by surgeons and other medical specialties. Therefore, a procedure considered an IR intervention in one center may be performed by a different specialty in another setting. Although the questionnaire used in our study was developed with consideration of national practice patterns, these differences should be taken into account when making international comparisons.

### Limitations

This study has several limitations that should be acknowledged. First, there is no dedicated radiology rotation within family medicine residency training, and radiology education is included only as an elective component. Second, the data collection was completed approximately three years ago, which may limit the extent to which the findings reflect current levels of interventional radiology awareness and clinical practice patterns. Third, the cross-sectional design captures knowledge and attitudes at a single point in time and does not allow for assessment of temporal changes or causal relationships. Fourth, the use of a self-reported online questionnaire may have introduced response bias, as participants may have overestimated or underestimated their level of knowledge. Fifth, although the questionnaire was developed with consideration of national practice patterns, differences in healthcare systems and access to interventional radiology services may limit the generalizability of the findings to family medicine residents in other countries. Sixth, the internal consistency of the 10-item knowledge assessment section was found to be low (Cronbach’s alpha = 0.51). Finally, although the sample size was adequate, participation was voluntary, which may have introduced selection bias, as residents with greater interest in or awareness of interventional radiology may have been more likely to participate.

### Clinical implications

The findings of this study suggest that interventional radiology (IR) education should be strengthened within family medicine residency training. Family physicians often serve as the first point of contact within the healthcare system and play a key role in referring patients to appropriate specialties; therefore, improving their level of knowledge regarding IR may contribute to timely and appropriate patient management. Incorporating structured IR rotations into residency curricula, or implementing targeted IR teaching sessions, short-term clinical observerships/rotations, and case-based interactive training, may enhance knowledge and confidence, thereby positively influencing referral practices and patient care. In addition, regular in-service training programs and multidisciplinary meetings within the framework of continuing professional development (such as case discussions and review sessions on referral pathways) may further improve awareness and appropriate referral behavior.

### Future research recommendations

Future studies should consider longitudinal designs to evaluate how IR knowledge evolves throughout residency and into clinical practice. Investigations involving larger, more diverse populations across different regions and healthcare settings would help to better generalize findings. Additionally, interventional studies assessing the impact of specific educational programs or rotations on knowledge and referral behaviours are warranted. Qualitative research exploring barriers to IR referral and knowledge acquisition could provide further insights for designing effective educational strategies. Finally, evaluating patient outcomes related to improved IR referral practices could further clarify the clinical significance of enhancing physician knowledge in this field.

## Conclusion

This study demonstrated that family medicine residents in Türkiye have a moderate level of knowledge regarding interventional radiology (IR). The finding that participants who had completed clinical rotations involving IR-related procedures achieved higher knowledge scores suggests that clinical exposure may enhance knowledge levels. The inclusion of radiology rotations and hands-on training within residency curricula may increase awareness of IR procedures and support appropriate referral practices.

## Supplementary Information

Supplementary Material 1.

## Data Availability

The survey instrument was developed based on a thorough review of the existing literature and was inspired by validated questionnaires from previous studies addressing similar topics. The final questionnaire was prepared collaboratively by two experts: SD, a family medicine specialist with 30 years of clinical experience, and MG, an interventional radiologist with 15 years of clinical experience. The questionnaire used in this study was developed by the authors based on previous literature and expert opinion (see Supplementary File 1).
