# Supplementary material for: Interventional radiology knowledge among family medicine residents in Türkiye: a national cross-sectional survey
Source: BMC Med Educ. 2026 Jul 11;26:1258. doi: 10.1186/s12909-026-09916-y (PMC13445882; doi:10.1186/s12909-026-09916-y)
Supplement: Supplementary file 1 [file 12909_2026_9916_MOESM1_ESM.pdf]

Questionnaire Used in the Study (English Version)

Data Collection Form

1. What is your gender?
( ) Female
( ) Male

2. How many years have you been practicing medicine?
………………..

3. Which year of your residency training are you currently in?
( ) 1st year
( ) 2nd year
( ) 3rd year
( ) 4th year or above

4. In which type of institution are you completing your residency training?
( ) University hospital
( ) Training and research hospital

5. Which residency training pathway are you enrolled in?
( ) Contracted Family Medicine Specialization (SAHU)
( ) Family Medicine Specialization (AHU)

6. Have you completed the rotations required during your residency training?
( ) Yes
( ) No

7. If your answer is No, which rotations have you not yet completed?
………………..

8. How would you describe your level of knowledge about interventional radiology?
( ) Good
( ) Moderate
( ) Poor

9. From where did you obtain your knowledge about interventional radiology?
( ) During medical school
( ) During residency training
( ) Medical articles, journals, or brochures
( ) Social media
( ) Colleagues
( ) Other: ………………..

10. Is there an interventional radiology unit in your hospital?
( ) No
( ) Yes
( ) I do not know

11. Interventional radiologists complete their specialty training in the following areas (please mark all that apply):
( ) Diagnostic radiology
( ) Image-guided interventions
( ) Surgical care

( ) Medical care
( ) All of the above

12. Have you or any of your relatives previously undergone an interventional radiology procedure?
( ) No
( ) Yes

13. Have you ever directly referred any of your patients to interventional radiology for an elective procedure?
( ) No
( ) Yes

Knowledge Questions

Which of the following procedures are performed by interventional radiology?
Please leave the question blank if you are unsure.

14. Performing biopsy or drainage procedures using imaging techniques
( ) True
( ) False

15. Cardiac angioplasty or stenting
( ) True
( ) False

16. Vascular angioplasty procedures
( ) True
( ) False

17. Lower extremity vascular bypass procedures
( ) True
( ) False

18. Interventional radiology can be used in the treatment of epistaxis
( ) True
( ) False

19. Uterine artery embolization can be used in the treatment of symptomatic uterine fibroids (myomas)
( ) True
( ) False

20. Interventional radiology procedures can be used for the treatment of breast fibroadenoma
( ) True
( ) False

21. Interventional radiology procedures can be used for the treatment of pelvic congestion syndrome
( ) True
( ) False
